# Chronic Diarrhea: A rare presentation of Hodgkin’s Lymphoma

**DOI:** 10.12669/pjms.38.8.5452

**Published:** 2022

**Authors:** Saira Furqan, Sajjad Ali Khan, Dania Ali, Kaleemullah Badini

**Affiliations:** 1 Saira Furqan, FCPS, Section of Endocrinology, Department of Medicine, Aga Khan University, Stadium Road, Karachi, Pakistan; 2 Sajjad Ali Khan, FCPS Fellow, Section of Endocrinology, Department of Medicine, Aga Khan University, Stadium Road, Karachi, Pakistan; 3 Dania Ali, MBBS. Research Associate, Section of Endocrinology, Department of Medicine, Aga Khan University, Stadium Road, Karachi, Pakistan; 4 Kaleemullah Badini, FCPS, Fellow, Section of Endocrinology, Department of Medicine, Aga Khan University, Stadium Road, Karachi, Pakistan

**Keywords:** Hodgkin’s Lymphoma, Chronic Diarrhea, Extra nodal GI involvement, Case Report

## Abstract

Hodgkin’s Lymphoma is a cancer affecting the lymphatic system. There are two subtypes of Hodgkin’s Lymphoma: Nodular lymphocyte predominant (NLPHL) and classical Hodgkin’s Lymphoma which has further four types. It has a bimodal distribution and is more common among males. The most common presentation is painless swelling in the neck, armpit or groin region. Associated Symptoms include night sweats, unintentional weight loss, fever, persistent cough or dyspnea. Involvement of the gastrointestinal tract is a rare occurrence. Diagnosis is based on hematological, radiological and histochemical studies. Here we report a rare presentation of a 47-year-old male who presented with symptoms of loose stools, weight loss and fever. CT scan and abdominal lymph node biopsy findings were consistent with a lymphoproliferative disorder Patient was being managed in the line of malabsorption syndrome with possible underlying malignancy but his condition deterioted before the histological diagnosis was confirmed. Thus, this report highlights the importance having a consideration for Hodgkin’s lymphoma in the management of chronic diarrhea.

## INTRODUCTION

Lymphomas are cancers originating in the White blood cells in our body, they are divided into Hodgkin and Non Hodgkin types.^1^ There are two subtypes of Hodgkin’s Lymphoma: Nodular lymphocyte predominant (NLPHL) and classical Hodgkin’s Lymphoma, classical Hodgkin’s Lymphoma has four subtypes Nodular Sclerosis HL, Mixed Cellularity HL, Lymphocyte rich HL and Lymphocyte depleted HL.^1^ NLPHL accounts for 5-10% and classical type accounts for 85% of the cases of Hodgkin’s Lymphoma.^2^ The disease peaks at two ages; in young adults and patients 55 years and older.^3^ Males are commonly affected, especially in the pediatric age group where 85% cases are males. Risk factors include EBV infection, autoimmune conditions and immunosuppressive states. Hodgkin Lymphoma may be familial as well.^4^ The most common presentation is painless swelling in the neck, armpit or groin region. Associated Symptoms include night sweats, unintentional weight loss, fever, persistent cough or dyspnea.^5^ FDG PET/CT of the chest, abdomen, and pelvis is the preferred imaging tool. Diagnosis is confirmed based on findings of biopsy from a lymph node or suspected organ.^4^

Combination chemotherapy is used for treatment of Hodgkin’s Lymphoma. Used combinations include ABVD (doxorubicin, bleomycin, vinblastine, and dacarbazine), Stanford V (a chemotherapy regimen consisting of mechlorethamine, doxorubicin, vinblastine, vincristine, bleomycin, etoposide, and prednisone), or BEACOPP (bleomycin, etoposide, doxorubicin, cyclophosphamide, vincristine, procarbazine, and prednisone) with radiotherapy.^6^ Gastrointestinal tract involvement in cases of Primary Hodgkin’s Lymphoma is an extremely rare occurrence.^7^ The overall prognosis of gastric Hodgkin’s Lymphoma is very poor, and between 45 to 60% of the patient’s dying within a year of diagnosis.^8^

Here we report a rare presentation of Hodgkin’s Lymphoma. The patient presented to our center and was diagnosed with Hodgkin’s Lymphoma based on radiological and Biopsy findings consistent with the disease. The medical record of the patient was reviewed extensively and relevant data recorded.

## CASE PRESENTATION

A 47-year-old male, weighing 80 kgs, with a previous history of exfoliative dermatitis and left leg deep venous thrombosis (DVT) presented with complaints of loose stools for the past two months (watery in nature, 15-20 episodes/day, not associated with nausea and vomiting, malenia or hematochezia and abdominal pain), Weight loss of five Kgs in the past two months (unintentional, decreased oral intake) and fever for the past 5 days (high grade, intermittent, no rigors and chills).

Prior to presentation to our center, patient had visited a local hospital for loose stools where he was given symptomatic treatment. Patient also consulted with a Gastroenterology specialist before presenting to our center. He had a previous one-week admission history at another private hospital before he presented to our center where extensive workup for his loose stools was carried out, however, the results of the testing were inconclusive.

Patient had a history of Hyper IgE syndrome and Atopic dermatitis. Family history was unremarkable for malignancy, tuberculosis or any chronic condition.

On presentation, patient was afebrile, tachycardiac with pulse of 120 bpm, respiratory rate of 16, sO2 96% and blood pressure 120/70 mmHg. General physical examination of the patient revealed pallor, oral ulcers and bilateral non pitting edema of the feet. Rest of the general physical and systemic examination was unremarkable. Baseline laboratory investigations were carried out along with relevant radiological investigations Laboratory findings are summarized in Table-I.

**Table-I:** Laboratory Investigations.

	Lab Parameters	4^th^ April	25^th^ April	29^th^ April	1^st^ May
Complete Blood Count	Hemoglobin (g/d)	11.2	10.3	11.6	9.1
	Leukocytes (10 ^9/L)	4.2	2.6	5.7	4.8
	PLT (10^9/L)	233	154	137	114
Renal Profile Electrolytes	BUN (mg/d)		18	60	71
	Cr (mg/d)	0.6	1.0	2.4	2.7
	Na (mEq/L)	134	136	137	140
	K (mEq/L)	2.6	3.4	3.9	3.5
	Cl (mEq/L)	100	106	105	104
	HCO3 (mEq/L)	24	20.5	14.3	11.5
	Ca (mg/d )			6.8	7.5
	Mg (mg/d)		2.1	2.4	2.5
	P04 (mg/d)			5.0	4.9
Liver Function Test	Albumin (g/dL)	2.4			
	TB (mg/d)	0.73	1.0		
	DB (mg/d)	0.3	0.9		2.2
	GGT (U/L)	186	274		2.1
	SGPT (U/L)	37	36		206
	SGOT (U/L)	49	59		34
	ALP (U/L)	308	400		82
	PT (Sec)	30.9	19.0		501
	INR	3.2	1.9		30.3
Infection Markers	CRP (mg/d)	84.64	109.83		3.2
	PCT (ng/ml)	0.18	5.86		

Esophagogastroduodenoscopy and Colonoscopy was unremarkable. CT chest, abdomen and pelvis with contrast revealed significant abdominopelvic lymphadenopathy with multiple subcentimeter mediastinal lymph nodes and splenomegaly.

Ultrasound guided core biopsy of enlarged lymph nodal mass along the left external iliac vessels was performed. Lymph node biopsy revealed tissue exhibiting features of CD30 lymphoproliferative disorder. In the differentials classical Hodgkin Lymphoma and Anaplastic Large Cell Lymphoma are included. However, considering the immunohistochemical profile, Classical Hodgkin Lymphoma is favored.

### Hospital course and outcome:

During the hospital stay patient had oligouria and became tachypneic and tachycardic because of deteriotong renal function so he underwent a single session of hemodialysis. Meanwhile, patient was being investigated for Malabsorption syndrome/Malignancy while the laboratory investigations and other relevant workup was awaited.

During the subsequent days, patient dropped GCS and became hypotensive for which he was started on inotropic support. His breathing became labored and he had to be intubated and shifted to the Medical Intensive care unit (ICU). During his ICU stay, patient had run asystole for which CPR was performed and return of spontaneous circulation (ROSC) was achieved after 20 minutes of CPR. A few hours following this episode, patient dropped pressures and bradycardic; no intervention was done considering the DNR status of the patient. Subsequently, patient expired.

## DISCUSSION

We present a case of a 47-year-old male diagnosed with Hodgkin’s Lymphoma on abdominal lymph node biopsy. While the most common presentation is painless lymphadenopathy and associated Symptoms include night sweats, unintentional weight loss, fever, persistent cough or dyspnea,^5^ our study patient presented with complaints of loose stools for the past 2 months along with associated symptoms of weight loss, swelling of the left leg and fever. This is a rare presentation of the disease. Patient was investigated and treated at our center before he expired. This unusual presentation of Hodgkin’s Lymphoma needs to be discussed so healthcare workers may become aware of the uncommon sites where the disease may occur.

Hematological and Biochemical workup of the patient was done. Initial results showed mild anemia and hypokalemia. Patient exhibited a decline in Hemoglobin, Platelets and White blood cells over the course of his hospital stay.

Contrast-enhanced CT scan, PET scan, endoscopy; both conventional and capsule and contrast radiography are used as initial radiological investigations for a suspected case of GI Lymphoma.^9^ We performed CT chest, abdomen and pelvis with contrast, which showed findings consistent with Lymphoproliferative disease. Endoscopy and colonoscopy of our patient was also performed, but the results were inconclusive.

Histopathology can be supplemented with Immunohistochemistry in cases where the diagnosis is not very clear. Immunohistochemical staining is now a routine investigation done for Lymphoma diagnosis. Commonly tested antibodies to rule in classical Hodgkin’s Lymphoma include CD3, CD20, CD15 and CD30. Classical cases of Hodgkin’s lymphoma are positive for CD30 and CD15. Similar findings were seen in case by Kilaru et al.^9^ Our case was positive for CD 30 but negative for CD15, similar findings were seen in presented by Jung et al.^8^ Typical CHL may have CD-15 negative immunohistochemistry in about one-third of the cases.^10^

Combination chemotherapy is used for treatment of Hodgkin’s Lymphoma. Used combinations include ABVD (doxorubicin, bleomycin, vinblastine, and dacarbazine), Stanford V (a chemotherapy regimen consisting of mechlorethamine, doxorubicin, vinblastine, vincristine, bleomycin, etoposide, and prednisone), or BEACOPP (bleomycin, etoposide, doxorubicin, cyclophosphamide, vincristine, procarbazine, and prednisone) with radiotherapy. Hodgkin’s Lymphoma is treated with combination chemotherapy, however, in our case the patient did not undergo any treatment as he expired within a few days of diagnosis.

## CONCLUSION

This was a rare presentation of Hodgkin’s Lymphoma. Such uncommon presenting features of the disease should be studied in more detail to prevent misdiagnosis and link the patient to treatment at the earliest.

### Authors’ Contributions:

**SF:** Provided supervision and reviewed the final manuscript.

**SAK:** Identified and prepared the manuscript is responsible and accountable for the accuracy or integrity of the work.

**DA:** Helped in preparing Abstract and writing the manuscript

**KB:** Helped in abstract and discussion writing.
